# Microscale sensor solution for data collection from fibre-matrix interfaces

**DOI:** 10.1038/s41598-021-87723-9

**Published:** 2021-04-16

**Authors:** Royson Dsouza, Paulo Antunes, Markus Kakkonen, Olli Tanhuanpää, Pekka Laurikainen, Farzin Javanshour, Pasi Kallio, Mikko Kanerva

**Affiliations:** 1 Faculty of Engineering and Natural Sciences, Tampere University, PO Box 589, 33014 Tampere, Finland; 2 Faculty of Medicine and Health Technology, Tampere University, PO Box 589, 33014 Tampere, Finland; 3 Fibrobotics Oy, Tampere, Finland; 4 Instituto de Telecomunicações, PO Box 3810-193, Aveiro, Portugal; 5 Physics Department and I3N, Aveiro University, Campus de Santiago, PO Box 3810-193, Aveiro, Portugal

**Keywords:** Techniques and instrumentation, Mechanical engineering, Lasers, LEDs and light sources

## Abstract

Especially the applications of fibrous composites in miniature products, dental and other medical applications require accurate data of microscale mechanics. The characterization of adhesion between single filament and picoliter-scale polymer matrix usually relies on the experiments using so-called microbond (MB) testing. The traditional MB test systems provide unitary data output (i.e., converted force) which is enigmatic in resolving the fracture parameters of multi-mode interface cracks. As a fundamental basis, the momentary reaction force and respective local strain at the location of a non-ambiguous gradient are needed for a mechanical analysis. In this paper, a monolithic compliant based structure with an integrated Fiber Bragg Grating (FBG) sensor is developed and analysed. The stiffness of the compliant structure is estimated by using mathematical and finite element (FE) models. Qualification experiments are carried out to confirm the functional performance: MB testing of synthetic (carbon and glass) and natural (flax) single filaments are successfully performed. Quasi-static and dynamic analysis of the MB testing is carried out by using the FE method to interpret the response of the compliant structure. The developed strain-sensing CBPM-FBG holder shows excellent sensitivity during the MB tests for both synthetic and natural filaments, even at a low filament diameters as low as $7\,\upmu \hbox {m}$, making the monolithic compliant structure the first instrument capable of force-strain data output for bonded filament-droplet specimens.

## Introduction

The major proportion of advanced engineering materials today consists of fibre-reinforced composites (FRCs) due to their specific, mainly density normalized, high strength and stiffness. Apart from the high strength and stiffness, they also possess attractive functional properties, such as corrosion resistance, electrical conductivity, thermal conductivity and directional properties (anisotropy) that can be tailored. FRCs’ can be tailored for a targeted application by tuning the three main constituents namely fibre, matrix and the interface between the fibrous reinforcement and the surrounding matrix. Fibres can consist of bundles of few to thousands of elementary fibres or filaments depending on the filament material and type. Although fibre volume fraction, matrix type and fibre direction are the fundamental building blocks of FRC’s in macroscale, certain functional properties as well as exact mechanical performance and damage evolution depend on the interface^1^. Due to the abundance of options for engineers, FRCs have been applied to most extreme applications, such as medical implants^2^, dental bridges^3^ and satellite enclosures^4^ to mention a few. The applications of natural fibre composites represent interesting alternatives to synthetic fibres, but also they have unique design challenges^5^.

**Figure 1:**
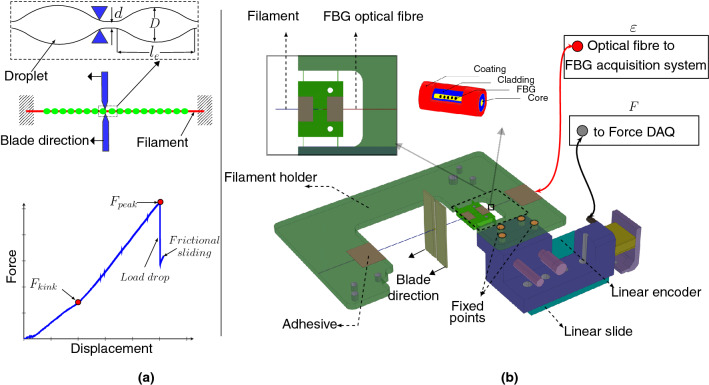
(**a**) The traditional MB test and related parameters. (**b**) The experimental setup of a MB test system with the CBPM-FBG filament holder concept. [(**a**) is generated using latex-pgf plot package, (**b**) is generated using Solidworks software, version 2018, https://www.solidworks.com/].

Interfacial damage at a micro level has been investigated for the past few decades to understand the damage evolution and failure at the matrix-reinforcement interfaces of FRCs. Various single fibre test methods, such as the pull-out test^6^, push-out test^7,8^ and microbond (MB) test^9–11^, are widely used but no specific standards exist^12^. In the MB test, picoliter-size resin droplets are deposited on single filaments. The single filament specimens are attached to a type of micromechanical tester. The droplets are pushed along the filament to mechanically break the tiny interface by using sharp-edged metal blades (see Fig. 1a). By far, this process is manual.

The practical embedded length ($l_e$) for MB test varies from $10$ to $200\,\upmu \hbox {m}$, resin droplet diameter (*D*) varies from $10$ to $180\,\upmu \hbox {m}$, the diameter of the fibre (*d*) varies from $6$ to $20\,\upmu \hbox {m}$, and the measured reaction forces (*F*) vary from 0–1 N^13,14^. To date, the device outputs include only force recordings and specimen or blade displacement from a sensor or the control signal (i.e., blade or grip area of the filament depending on the test method)^15^. Naturally, the control signal is independent of the filament and matrix and, therefore, cannot sense the specimen’s behaviour. Due to the small size, a displacement sensor for a micrometer-length scale interface cannot be fixed to the specimen but to the test machine structure—being prone to various lags and compliance^16,17^. The MB test is also prone to various sources of errors, such as load measurement, device optics and resin curing, which can lead to high scattering in test results^18^. Through the years, these drawbacks have prevented researchers from producing reliable interface data and highly optimized composite applications.

Integrated optical sensors are potential to solve the interfacial measurements yet several challenging aspects are to be considered: (1) For a laser-excited Fibre Bragg grating sensor (FBG), the location of the sensing point must be exactly in line with the specimen [filament and droplet(s)]. (2) Any compliant mechanism^19^ that can transmit the specimen’s deformation to the strain sensor, must possess a high resilience. (3) The inertia and dissipation in the measurement system must be minimized for the sensor to follow the dynamics of the specimen during testing. The strain sensitivity enhancement using compliant flexural hinges have demonstrated a high precision and accuracy in strain measurements^20^. The flexural hinges provide high motion resolution, negligible cross-coupling errors and fast response and make it a superior candidate for a system with micro-scale droplet-filament specimens and FBG sensor technology for measurements. FBGs can provide long-term, stable measurements with essentially zero drift and fatigue durability. However, they are not as robust as strain gauges, for example. The optical path (fibre) to the sensor (FBG) must be clean and perfect for error-less and strong laser signal. Therefore, all connections should be minimized; practical connections of optical fibres require occasional cleaning and care when re-connected. At least one connection between the specimen of measurements and the data acquisition is always needed. The applied connector element must be kept well aligned and intact. If tests are carried out in an outdoor environment or an environmentally controlled chamber with air circulation, the air fluctuation and related temperature variations in each FBG need to be balanced by a separate FBG for this function.

To solve the above challenges of measurement and reveal the true behaviour of filament-polymer interfaces, an especial compound basic parallelogram mechanism (CBPM) with an integrated FBG sensor is studied here. The first ever functional specimen holder is numerically simulated to understand the static and dynamic system response to a physically complex interface damage evolution. Comprehensive sensitivity and dynamic (cyclic) tests are carried out to verify the device performance in reality and numerical analysis of the tested interfaces. The actual qualification is finally performed by coupling the new system with a MB test machine and by testing carbon, glass and flax filament specimens at an extreme accuracy.

## Results

### CBPM integration and geometry effects

The strain sensing by using an optical fibre-located FBG is tailorable, small in size and integrable to a flexible filament, i.e. the directional sensitivity can be high. The FBG is based on the periodic perturbation of the refractive index along the direction of the fibre. Theoretically, an FBG sensor does not have an accuracy limit for the strain detection but the practical accuracy is limited by the signal analysis (Fourier transform) of the reflected laser. Therefore, the deformation experienced by the sensor can be scaled (down) during its operation. For the MB tests as final application target, the solution for re-mounting of test filament and the adjustment of sensor deformation (i.e., adjustment of the filament holder’s compliance) can be achieved by introducing a CBPM at the fixed region of the laser routing (optical fibre). Here, the precise alignment of the slender (flexible) optical fibre is performed by a slight tension while fixing the optical fibre to its place on the holder basis. Figure 1b represents the new CBPM based filament holder and an integrated FBG sensor along the optical fibre; the arrangement in the FIBRObond tester^18^ (Fibrobotics Oy, Finland) is illustrated as an operative application when using the new CBPM-FBG filament holder.

**Figure 2:**
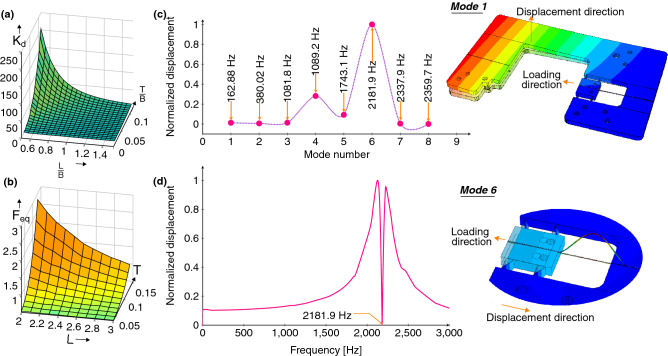
(**a**) The influence of geometrical parameters on $K_d$; (**b**) Geometrical value selection based on $F_{eq}$; (**c**) Eigen frequencies of the new device; (**d**) The modal analysis of the system for mode six (2181.9 Hz). [ Graphs are generated using latex-pgf plot package, FE models for (**c**) and (**d**) are generated using Abaqus Standard/Explicit software, version 2017, https://www.3ds.com/products-services/simulia/products/abaqus/].

The CBPM unit of an integral filament holder can be designed by using mathematical modelling (see the section of “Methods”) with an approximate geometry. According to the model, as the strip’s length to width ratio (*L*/*B*) increases, the stiffness damping factor ($K_d$) sharply decreases whereas the factor’s value ($K_d$) increases along with the strip’s thickness to width ratio (*T*/*B*), as shown in Fig. 2a. The values of *L* and *T* are to be selected based on the maximum force (i.e., 1.0 N here) and the lowest $K_d$ to make sure that the sensitivity of the compliant system is suitably high. According to Fig. 2b and the manufacturing aspects (mainly accuracy limits of fabrication techniques), the value of *L* and *T* shall be taken to be 2.6 mm and 0.1 mm, respectively, for the instrument manufacturing of this study.

The exact compliance for the final geometry of the CBPM-FBG filament holder must be precisely predicted by the finite element analysis (FEA) (see the section of “Methods”). The first eight modes of natural frequencies of the modelled filament holder are shown in Fig. 2c as a function of displacement along the droplet-fibre specimen’s loading direction. It is seen that the mode six leads to the maximum amplitude of deformation and it occurs along the direction of the load. This can be observed in Fig. 2c,d. In the following, a steady-state dynamic analysis and a frequency sweep was applied from 0 to 3000 Hz and the resonant frequency of 2181.9 Hz was covered. This very closely corresponds to the mode six behaviour (see Fig. 2d) and is far away from any known droplet-filament response.

### CBPM-FBG behaviour in tensile loading

**Figure 3:**
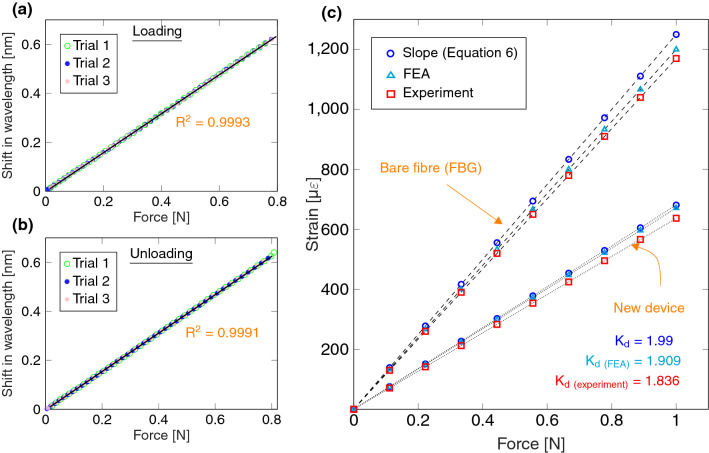
(**a**) The indicated FBG wavelength shift as a function of measured tensile force during loading; (**b**) the unloading ramp of a tensile test; (**c**) the numerically predicted and experimentally measured force-strain responses. [The graphs are generated using latex-pgf plot package].

Figure 3a,b shows the graphs of the reflected (from FBG) laser’s Bragg wavelength shift as a function of recorded strain for both loading and unloading ramps ($R^2=0.999$). The sensitivity of the FBG sensor was found to be 0.7958 pm/mN for the applied loading ramp and 0.786 pm/mN for the unloading ramp. The 0.9% decrease of sensitivity for the unloading is due to the slight dynamic movement and spring-back of the CBPM/stage.

Figure 3c shows the graphs of measured force as a function of strain from (I) mathematical, (II) FEA, and (III) experiments. Here, the experimental results for the *bare optical fibre* (with FBG) were obtained using the micro-tensile setup, without the presence of frame/CBPM, to simply load the FBG sensor. It can be seen that a 4.2% error in $K_d$ (giving displacement at the FBG location) is observable between Eq. 6 and FEA due to the 3D effects of exact geometry, such as rounded corners not considered in the mathematical model. In turn, a 3.8% error can be observed between the experiments and FEA and this indicates that the dynamic loading needs to be analyzed—in the event of dissipation, nonlinearity included in the discrepancy could lead to recorded strain drift during cyclic testing.

**Figure 4:**
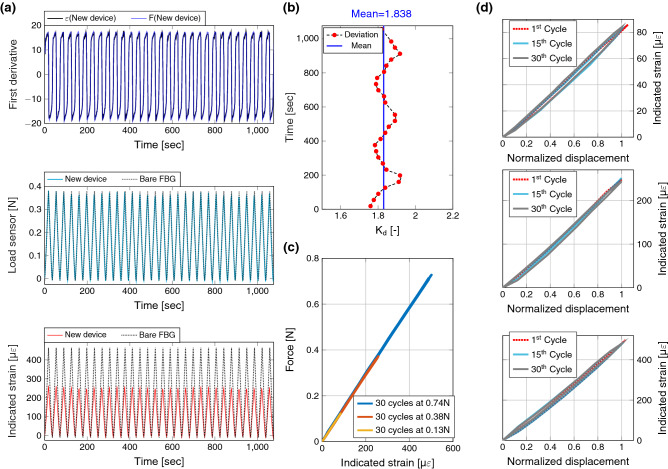
(**a**) The measured strain for 30 cycles for the load level of 0.38 N; (**b**) the measured force for the new CBPM-FBG holder and bare FBG; (**c**) the first derivative of the measured force and strain; (**d**) the standard deviation of derived $K_d$ (based on input and FBG strain) during three typical cycles. [The graphs are generated using latex-pgf plot package].

Figure 4a represents the strain and force responses for simple FBG and the new device. The first derivatives indicate a perfect synchronous system (strain and force) of the designed system. $K_d$ was computed by taking the ratio of bare optical fibre strain to the measured strain from the structure for over 30 cycles at 0.74 N ‘peak load’. A standard deviation of 0.0431 in the overall $K_d$ was found as indicated in Fig. 4b. Figure 4d shows the measured strain as a function of test machine displacement for three different intermediate loading cycles. Hysteresis can be observed that is evident from the curves forming loops instead of lines. Figure 4c shows the measured force as a function of strain for 30 cycles and the three intermediate (peak) load levels. No hysteresis was observed for the strain-force space. This indicates that the hysteresis arises from the test machine and not from the developed CBPM-FBG holder.

### Functionality for the coupling of the MB test machine to CBPM-FBG holder

**Figure 5:**
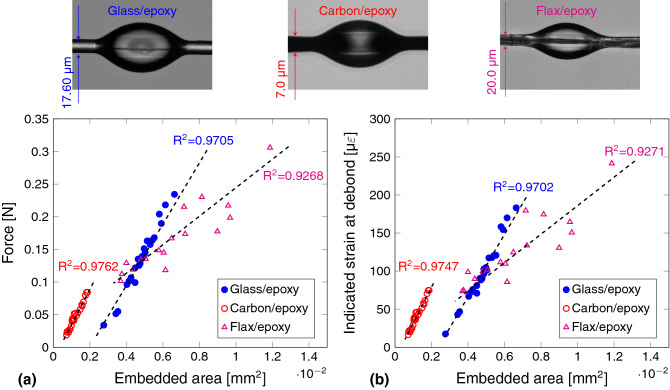
Systematic, serial MB test results for glass, carbon and flax filament batches (epoxy droplets) in terms of (**a**) the measured force; (**b**) the measured (FBG) strain by using the CBPM-FBG specimen holder. [The graphs are generated using latex-pgf plot package].

Figure 5 shows the variation of force and strain output as a function of droplet embedded area ($\pi d_f l_e$). The embedded length ($l_e$) of the droplet and filament diameter ($d_f$) are measured for each of the droplet before a MB test. An excellent agreement of $R^2 \succ 0.97$ is observed for the glass/epoxy and carbon/epoxy samples whereas flax/epoxy samples exhibit $R^2 \succ 0.92$. Linearity in the failure load-embedded area typically is understood as high-quality MB testing in the current literature of MB testing^18^.

**Figure 6:**
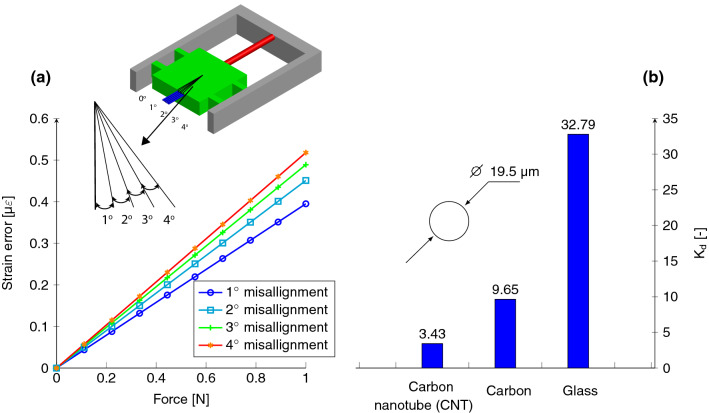
FEA to determine the predicted solutions of: (**a**) error in the FBG strain when a filament is incorrectly fixed to the stage: $\upvarepsilon _{ideal}-\upvarepsilon _{mis}$; (**b**) the $K_d$ values for different types (material) of filaments for a filament diameter of 19.5 $\upmu \hbox {m}$ (filaments are modelled as linear elastic with modulus of 600 GPa^21^, 238 GPa^22^ and 86 GPa^23^ for CNT, carbon and glass, respectively). [The graphs are generated using latex-pgf plot package].

During the MB tests performed, the droplet-filament specimen in a test is glued onto the stage. This gluing process is typically a manual process and might result in the misalignment of the filament. FEA was carried out for four misalignment levels (maximum misalignment of $4^ \circ$) and compared to the perfectly aligned filament as shown in Fig. 6a. Due to the four CBPM legs (’hinges’), the FBG stretches well despite the misalignment; the parallel hinges on the sides of the compliant mechanism ensure that the errors due to the misalignment are negligible (0.064% error at $4^\circ$ misalignment when compared to $0^\circ$ align strain). This produces a high through output of uniaxial load on the FBG sensor, which results in the elimination of errors caused due to mechanical handling of the droplet-filament specimen. The variety of fibres existing, with different material properties, and also different diameters make it necessary to scale the FBG strain to determine the strain of the filament being tested. The elastic (Young’s) modulus plays the major role in determining the $K_d$ per filament material. FEA was carried out to simulate the value of $K_d$ for different types of filaments as well as different fibre diameters (here glass, carbon and carbon nanotube (CNT) filaments). The computed specific values (for diameter of $19.5\,\upmu \hbox {m}$) are shown in Fig. 6b. The $K_d$ per filament material vary from 3.43...32.8 meaning that for exact (engineering) strain results, the Young’s modulus and diameter of the test filament must be known—for typical MB tests, the diameter is always (optically) determined and the modulus is given by the manufacturer.

### Numerically resolved high-resolution interfacial performance

**Figure 7:**
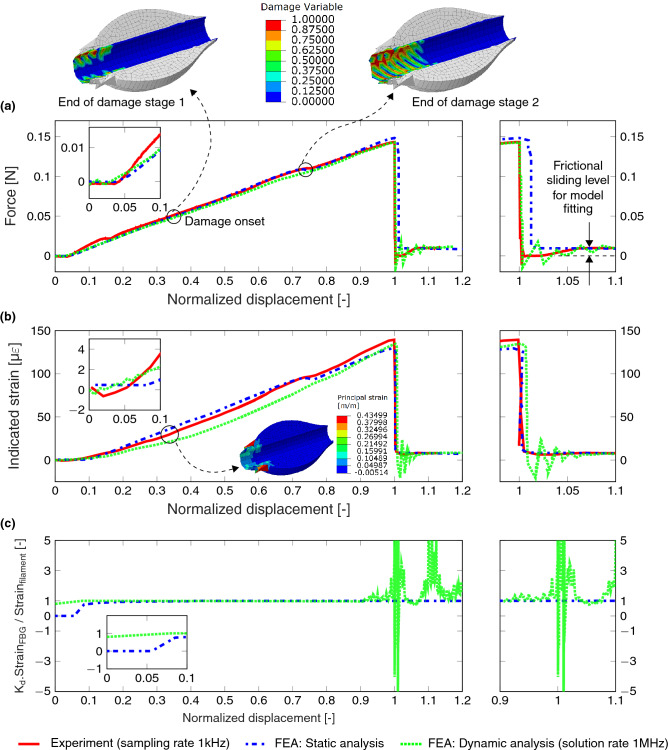
Different output results from FEA and experimentation: (**a**) the force as a function of test progress; (**b**) the strain (FBG) as a function of test progress; and (**c**) the ratio of FBG strain to filament strain based on FEA; test progress given by normalized blade displacement. $K_d$ = 32.79 (glass filament). The experimental force data has sampling rate of 1 kHz and the strain data 50 Hz. [FE insets for (**a**) and (**b**) are generated using Abaqus Standard/Explicit software, version 2017, https://www.3ds.com/products-services/simulia/products/abaqus/, the graphical plots are generated using latex-pgf plot package].

The detailed 3D FE model setup is described in the previous work^24^ and in the section of “Methods”. In this study, a frictional coefficient of 0.35 was fitted between the droplet and filament (the fitting’s target level after debond, see Fig. 7a). The comparison of experimentally recorded data with the FE results from (I) static (‘standard’) and (II) dynamic (‘explicit’) analysis is shown in Fig. 7. Up to the full debond, the magnitude of kinetic energy (FEA) was observed much lower than that of the internal energy, that validates the design of CBPM-FBG coupling for MB tests. It is also seen that there is a sudden increase in kinetic energy as well as frictional energy after the complete interfacial debond—this accuracy for the after-debond dynamics has not been available with current, traditional measurement systems. The small-scale vibrations in the filament, resolved by the dynamic FEA, are clearly observable but partly dissipated in the real CBPM-FBG system or frequency is beyond the test sampling rate (see Fig. 7c). The interfacial damage at two different locations is shown in Fig. 7a and the corresponding principal strain field at damage onset is shown in Fig. 7b. The two locations of damage clearly represent the state of the interfacial damage at the end of stage 1 and 2 respectively as presented in the previous work^24^.

## Conclusions

Advanced composites rely on the optimum combination of matrix properties, reinforcement performance and the interface between the two components. The characterization of the interface requires micro-meter scale interfacial loading and sensitive force-strain measurements. This work analyzed and qualified a completely novel system where the strain sensing by using a tailored FBG sensor in an optical fibre is combined with an titanium-integrated CBPM stage for deformation scaling and test specimen replacements. The effects of the CBPM’s geometry were surveyed using a mathematical modelling and the detail design was simulated with a finite element model. The scaling that is governed by a stiffness damping factor was determined with values of 9.65 and 32.79 for carbon and glass filaments (diameter $19.5\,\upmu \hbox {m}$), respectively. Any non-linearity with dissipation potential to data drift were found negligible for quasi-static (sensitivity of 0.796 pm/mN) and cyclic loading up to more than 30 repetitive cycles. For the first time ever, force-filament strain data was systematically collected for droplet-filament specimens at a sampling rate of $\ge 50\,\hbox {Hz}$ when the integral FBG-CBPM specimen holder was operated in the Fibrobond MB tester; three different material systems were analyzed. FEA of the MB testing with glass filaments and epoxy droplets enabled fitting and exact interfacial CZM-based debond model and frictional sliding with a friction coefficient of 0.35.

## Methods

### CBPM-FBG preparation

The new CBPM-FBG filament holder (its chassis) is made of Ti6Al4V [Young’s Modulus (E) of 114 GPa, Poisson’s ratio ($\nu$) of 0.342] class of alloy due to its high resilience [$3232\,\hbox {N}/\hbox {mm}^2$]. In practice, the only means to prepare the CBPM precisely is to use the Wire Electro Discharge Machining (WEDM) technique (here: by Mectalent Oy, Finland). The optical fibre [$\hbox {E}=70\,\hbox {GPa}$, $\nu =0.2$^24^] and the carved FBG (sensor) are bonded onto the remaining space between the outer strips of the CBPM and the filament mounting stage (see Fig. 1b). The silica FBG sensor itself was prepared with a device-specific (here: by Instituto de Telecomunicações, Aveiro, Portugal). A step index single-mode glass fibre (GF1, $\hbox {Nufern}^{\textregistered }$) was selected to conduct the laser signal in/out via the core with a diameter of $10\,\upmu \hbox {m}$. The cladding in the selected fibre has the (outer) diameter of $125\,\upmu \hbox {m}$. By using the phase mask method, a 3.0 mm-long FBG grating is inscribed into the fibre^25^. A W3/1050 series FBG interrogator (Smart $\hbox {Fibres}^{\textregistered }$) is used for flashing and reading the sensor. This interrogator has a wavelength range of 1510–1590 nm and an accuracy of $\pm 0.0006\,\hbox {nm}$. The interrogator was controlled using a remote interface W3 WDM (version 1.04) at a sampling rate of 50 Hz.

For the MB test machine used here, the force data is measured with a load cell of 1 N (Futek, US), which is connected to a linear slide. The slide in-turn is connected to an absolute linear encoder (Numerik Jena, Germany). The main advantage of the encoder connected to the slide is to measure elastic deformation of the load cell during loading. A data acquisition device NI-6003 (National Instruments, US) is used to record the force signal at a 1 KHz sampling rate. Microtome blades of a type R35 with a thickness of 0.254 mm (Feather, Japan) are used to excite loading to the droplet on the filament (test per individual droplet). The blade movement along the filament long axis is controlled with a M-111-1DG1 DC motor operated with a C-863 controller (Physik Instrumente, Germany). The FBG signal (strain indication) is zeroed before each measurement and so is the load cell of the testing device. Before any series of measurements, the load cell is initially calibrated (maximum error in force $=$ signal voltage $\times (\pm 0.001182)$ N). The experiments are carried out in (constant) ambient laboratory conditions ($20.4 \pm 0.04\;^{\circ }\hbox {C}$, $27.3 \pm 0.04$ %RH, statistics for a week).

In this study, for the particular characterization of the CBPM mechanism, the blades are momentarily replaced by a tensile mechanism in which the tensile stage is displaced until the applied load reaches 0.8 N. The force and strain data in general are recorded simultaneously using a defined time stamp on the systems (the Fibrobotics test machine and laser interrogator). Cyclic tensile tests are also performed with the tensile setup (see Fig. 10a) for a range of load levels (0.74 N, 0.38 N and 0.13 N) and 30 cycles per each level.

### Mathematical model of the compliant structure

**Figure 8:**
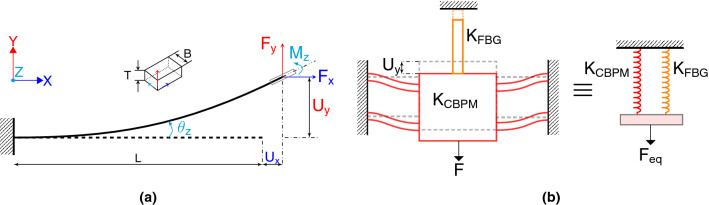
(**a**) Deformation of a flexural beam; (**b**) deformation of the four-arm CBPM.

The important criterion to be incorporated in the design of the filament holder is the parallel movement of the filament mounting stage and the droplet-filament specimen. In the design phase, the CBPM mechanism’s geometry is surveyed (see Fig. 8b) by using a non-linear, closed-form mathematical model of the CBPM. The four flexural strips of the CBPM are considered as long slender and planar beams based on the Euler/Bernoulli assumption. A single beam is modelled first to obtain a closed form solution based on the beam constraint model^26^. The structural modelling of the single flexural beam and deformation of the CBPM are illustrated in Fig. 8a,b, respectively. The beam constraint model for the transverse end load displacement relation is given by^26^:

1 $$\begin{aligned} \left[ \begin{array}{c} \frac{F_{y}L^{2}}{EI_{zz}} \\ \frac{M_{z}L}{EI_{zz}} \end{array}\right] =\left[ \begin{array}{cc} k_{11}^{(0)} &{} k_{12}^{(0)}\\ k_{21}^{(0)} &{} k_{22}^{(0)} \end{array}\right] \left[ \begin{array}{c} \frac{U_{y}}{L}\\ \theta _{z} \end{array}\right] +\frac{F_{x}L^{2}}{EI_{zz}}\left[ \begin{array}{cc} k_{11}^{(1)} &{} k_{12}^{(1)}\\ k_{21}^{(1)} &{} k_{22}^{(1)} \end{array}\right] \left[ \begin{array}{c} \frac{U_{y}}{L}\\ \theta _{z} \end{array}\right] . \end{aligned}$$

The dependence of the axial displacement on transverse displacement is given by^26^:

2 $$\begin{aligned} \frac{U_{x}}{L}= & {} \frac{1}{k_{33}}\frac{F_{x}L^{2}}{EI_{zz}}+\left[ \begin{array}{cc} \frac{U_{y}}{L}&\theta _{z}\end{array}\right] \left[ \begin{array}{cc} g_{11}^{(0)} &{} g_{12}^{(0)}\\ g_{21}^{(0)} &{} g_{22}^{(0)} \end{array}\right] \left[ \begin{array}{c} \frac{U_{y}}{L}\\ \theta _{z} \end{array}\right] \nonumber \\&+\frac{F_{x}L^{2}}{EI_{zz}}\left[ \begin{array}{cc} \frac{U_{y}}{L}&\theta _{z}\end{array}\right] \left[ \begin{array}{cc} g_{11}^{(1)} &{} g_{12}^{(1)}\\ g_{21}^{(1)} &{} g_{22}^{(1)} \end{array}\right] \left[ \begin{array}{c} \frac{U_{y}}{L}\\ \theta _{z} \end{array}\right] . \end{aligned}$$

Here, *E* is the Young’s modulus of the beam material, $F_x$ and $U_x$ are the axial end load and displacement, respectively, $F_y$ and $U_y$ are the transverse end load and displacement, respectively, $M_z$ is the moment, $I_{zz}$ is the second moment of area about the Z-axis, *L* is the length of the beam (strip) and $k_{ij}$ and $g_{ij}$ are the characteristic coefficients for a simple beam modelling a strip. A detailed derivation of the equations and the values of the characteristic coefficients are presented in Supplementary information 2.

Here, all loads, displacements and stiffness terms can be normalized with respect to the beam parameters as follows:

$$\begin{aligned}&\frac{F_{x}L^{2}}{EI_{zz}}=f_{x};~~~~\frac{F_{y}L^{2}}{EI_{zz}}=f_{y};~~~~ \frac{M_{z}L}{EI_{zz}}=m_{z}\\&\frac{U_{x}}{L}=u_{x};~~~~\frac{U_{y}}{L}=u_{y}; ~~~~\frac{T}{L}=t;~~~~k_{33}=\frac{12}{t^{2}}. \end{aligned}$$

By substituting $u_x=0$, $\theta _z=0$, multiplying the transverse end load with a factor of 4 (due to four flexible beams), and by substituting the values of the characteristic coefficients of the beam (tabulated in Table 1 of Supplementary information 2) we arrive to:

3 $$\begin{aligned} f_{x}= & {} \frac{6~u_{y}^{2}}{\frac{10}{k_{33}}+\frac{u_{y}^{2}}{70}} \end{aligned}$$

4 $$\begin{aligned} f_{y}= & {} u_{y}(12+1.2f_{x}). \end{aligned}$$

The stiffness in transverse direction can be calculated by substituting Eqs. (3) in (4) and rewriting the equation:

5 $$\begin{aligned} {K}_{{CBPM}}=\left( 48+\frac{5.76u_{y}^{2}}{\frac{10}{k_{33}} +\frac{u_{y}^{2}}{70}}\right) \frac{EI}{L^{3}}. \end{aligned}$$

Figure 8b shows the entire holder system wherein the CBPM is coupled with an FBG (optical fibre). There are two different stiffness mechanisms acting on the system and they can be reduced to equivalent parallel springs as shown in Fig. 8b. The axial stiffness of the optical fibre ($K_{FBG}$) is well known and the stiffness of the CBPM is calculated using Eq. (5). Hence, the stiffness damping factor for the system can be arranged as follows:

6 $$\begin{aligned} K_{d}=\frac{K_{CBPM}+K_{FBG}}{K_{FBG}}. \end{aligned}$$

### Finite element modelling and MB test simulation (Standard/Explicit)

A full 3D finite element model with the precise geometry of the final CBPM-FBG filament holder was developed using the software Abaqus Standard/Explicit 2017 (Dassault Systèmes, France). The glass filament, optical fibre, filament holder and adhesive are modelled as linear elastic materials and blades as rigid body.

**Figure 9:**
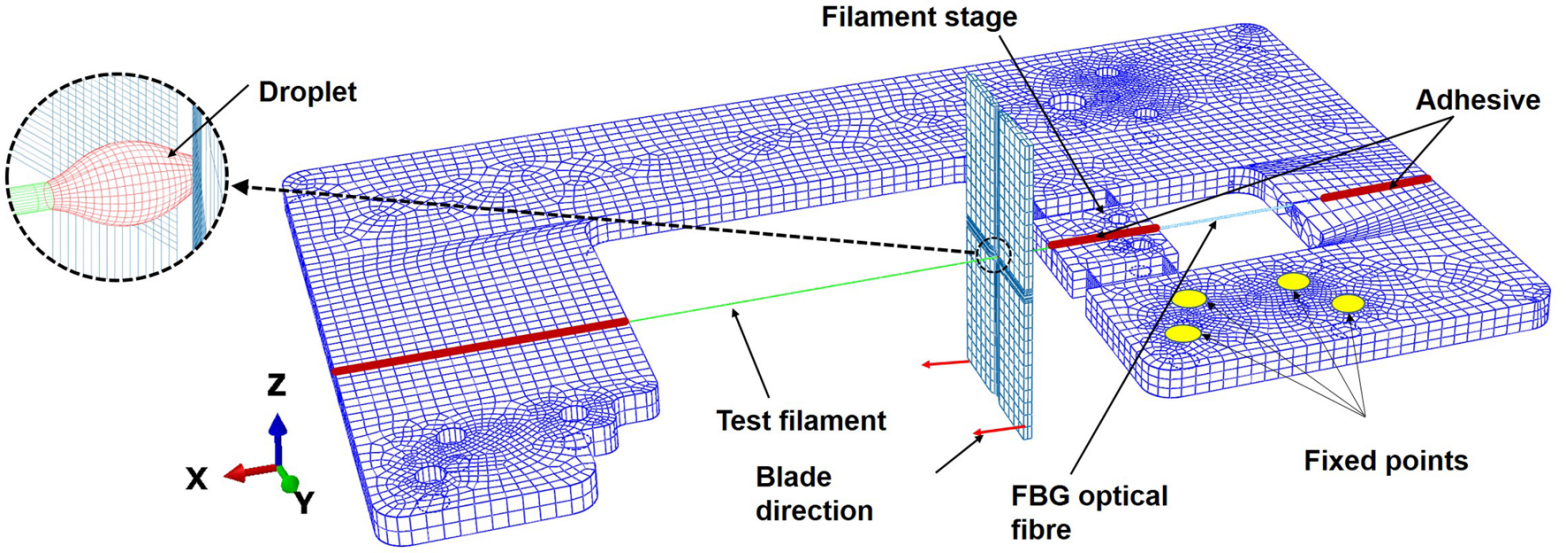
(**a**) FE model of the MB test system. An element type of 8-node linear brick (C3D8R) is used for all parts of the FE model. [This figure is generated using Abaqus Standard/Explicit software, version 2017, https://www.3ds.com/products-services/simulia/products/abaqus/].

**Table 1:** The values of the material constants used in the FE modelling.

Part	Modulus (GPa)	Poisson’s ratio (–)	Coefficient of thermal expansion ($\times 10^{-6}1/^{\circ }\mathrm {C}$)
Optical fibre^24^	70	0.2	–
Chassis (Ti6Al4V)	114	0.342	–
Epoxy^27^	3.2	0.35	71
Glass filament^23^	86	0.17	4.1
Adhesive	1.6	0.29	–
Blades (stainless steel alloy)	220	0.29	–

The epoxy droplet is treated as elastic–plastic wherein the material is linear-elastic uptill 60 MPa followed by plastic strain evolution (0%, 60 MPa; 0.2%,70 MPa) with the kinematic hardening conditions. The values of the material constants are presented in Table 1. The ‘fixed’ attachment points of the filament holder were constrained in all DOF’s, as shown in Fig. 9. The ends of the test filament were constrained on the filament holder’s stage using an adhesive block (the tie constraint at element interfaces). A section of elements was chosen on the FBG fiber having their characteristic length the same as the real FBG sensor—strains were recorded in this section during each simulation. A displacement was applied along the direction of the filament long axis ($U_y$) and corresponding force and strain data were recorded during simulation (see Fig. 3). The dynamic FE model was used to investigate the dynamic behaviour of the new filament holder.

### Qualification experiments

**Figure 10:**
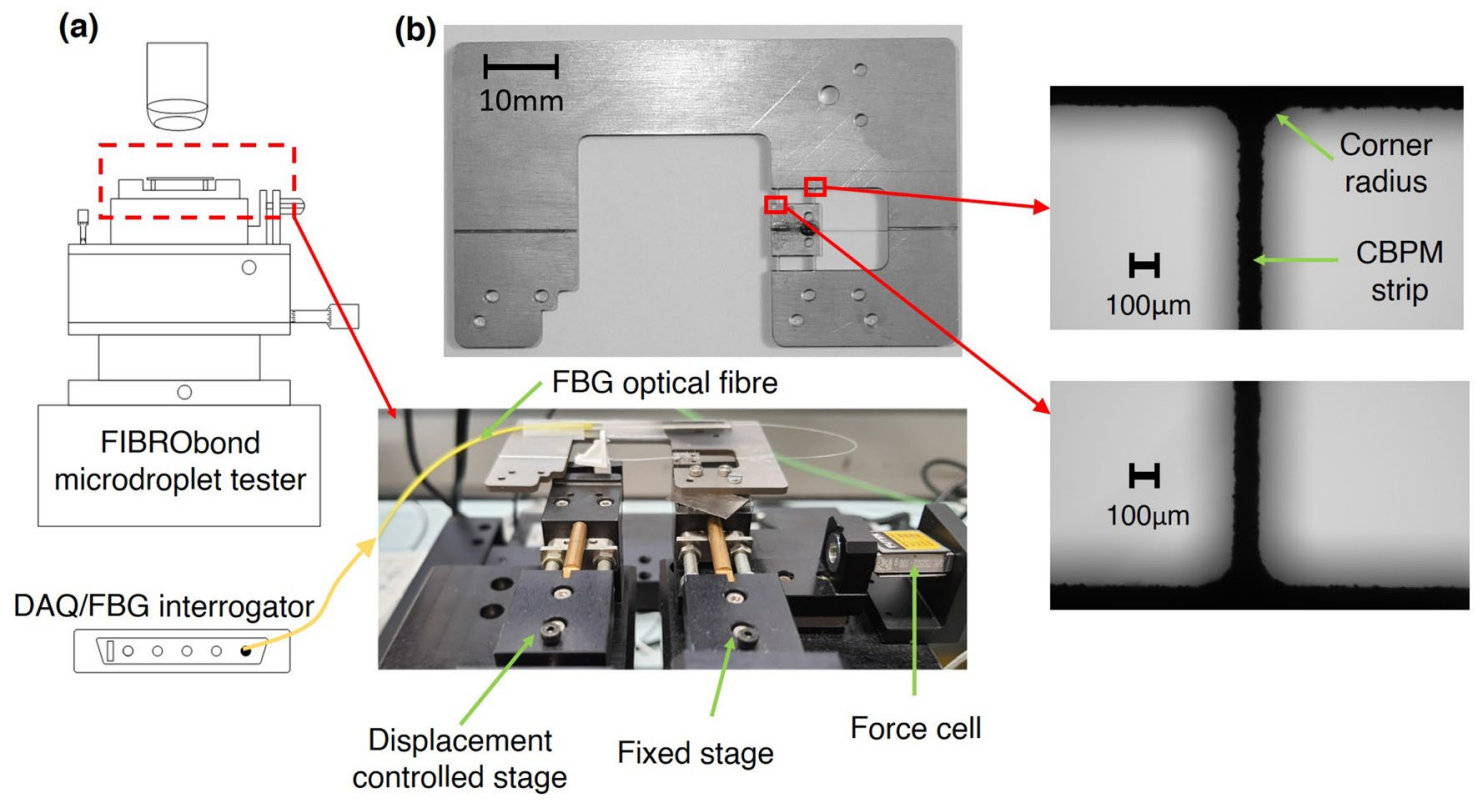
(**a**) Experimental tensile setup; (**b**) CBPM-FBG filament holder prepared for tests.

MB experiments were carried out by using the CBPM-FBG filament holder (see Fig. 10b) installed in the commercial MB test device as described in Fig. 1. The microdroplets were prepared by a specific^28^ resin dip method using the mixture of Araldite 5052 as resin and Aradur 5052 as hardener (provided by Hunstman Corporation, US) with a mixing ratio of 100/38 (mass/mass). The droplets were cured at $23\;^\circ \hbox {C}$ for 24 h followed by a post curing phase for eight hours (at $80\;^\circ \hbox {C}$). The droplets were deposited on carbon filaments—Toray T300 (provided by Toray Composite Materials, US; E= 140 GPa^29^) having a diameter of $7\,\upmu \hbox {m}$, glass filaments—HiPer-tex W 2020 glass fibre (provided by Ahlstrom-Munksjö Glass-Fibre Oy, Finland; E= 86 GPa^23^) of a diameter of $17.60\,\upmu \hbox {m}$ and also on flax filaments (provided by Bcomp, Switzerland; E= 61 GPa^30^) having an average diameter of $20\,\upmu \hbox {m}$. It is important to note that, as the test filament diameter increases, droplets of larger volume will be deposited on the filament—this typically leads to higher test loads as well. Thirty droplets were tested for each filament material.

## Supplementary Information

Supplementary Information.

Supplementary Video.

## Data Availability

Materials and data are available from the corresponding authors.
