# Supplementary material for: Microscale sensor solution for data collection from fibre-matrix interfaces
Source: Sci Rep. 2021 Apr 16;11:8346. doi: 10.1038/s41598-021-87723-9 (PMC8052447; doi:10.1038/s41598-021-87723-9)
Supplement: Supplementary file 1 — Supplementary Information. [file 41598_2021_87723_MOESM1_ESM.pdf]

Microscale sensor solution for data collection from fibre-matrix interfaces
Supplementary information

Dsouza, R[a,], Antunes, P[d,e], Kakkonen, M[b,c], Tanhuanpää, O[b,c], Laurikainen, P[a], Javanshour, F[a], Kallio, P[b], Kanerva, M[a]

[a] *Tampere University, Faculty of Engineering and Natural Sciences, PO Box 589, FI-33014 Tampere, Finland*
[b] *Tampere University, Faculty of Medicine and Health Technology, PO Box 589, FI-33014 Tampere, Finland*
[c] *Fibrobotics Oy, Finland*
[d] *Instituto de Telecomunicações, PO Box 3810-193, Aveiro, Portugal*
[e] *Physics Department and I3N, Aveiro University, Campus de Santiago,PO Box 3810-193, Aveiro, Portugal*

Supplementary information 1. FBG sensing principle

Fig. 1 demonstrates the working principle of a Fibre Bragg grating (FBG). Typical optical fibres (for FBGs) consist of an inner core, outer cladding and outer coating. For signal, an FBG is a periodic modulation of the refractive index along the fibre core. When the fibre stretches by the means of mechanical loading, the grating period and refractive index change from Λ to Λ' and n_{eff} to n'_{eff}, respectively, as shown in Fig. 1. The Bragg wavelength of the strained optical fibre is given by the first order Bragg condition:

$$\lambda_b = 2n'_{eff}\Lambda' \tag{1}$$

where λ_b is the Bragg wavelength, n'_{eff} is the fibre core refractive index and Λ' is the grating period of the fibre.

For a strain change of $\Delta\varepsilon$, the corresponding reflected wavelength shift is given by [1]:

$$\frac{\Delta\lambda}{\lambda_b} = (\alpha + \beta)\Delta T + (1 + P_e\Delta\varepsilon) \tag{2}$$

where $\Delta\lambda$ is the wavelength shift, α is the thermal expansion coefficient of the fibre, β is the thermo-optical coefficient and P_e is the photo elastic coefficient of the fibre. It is seen from Eq. 2 that the wavelength shift of FBG is linear per axial strain change, at a constant

temperature. In the current study, all the investigations are performed at a (constant) room temperature. After knowing the refractive index, the use of optical fibres (FBG) does not require any further calibration.

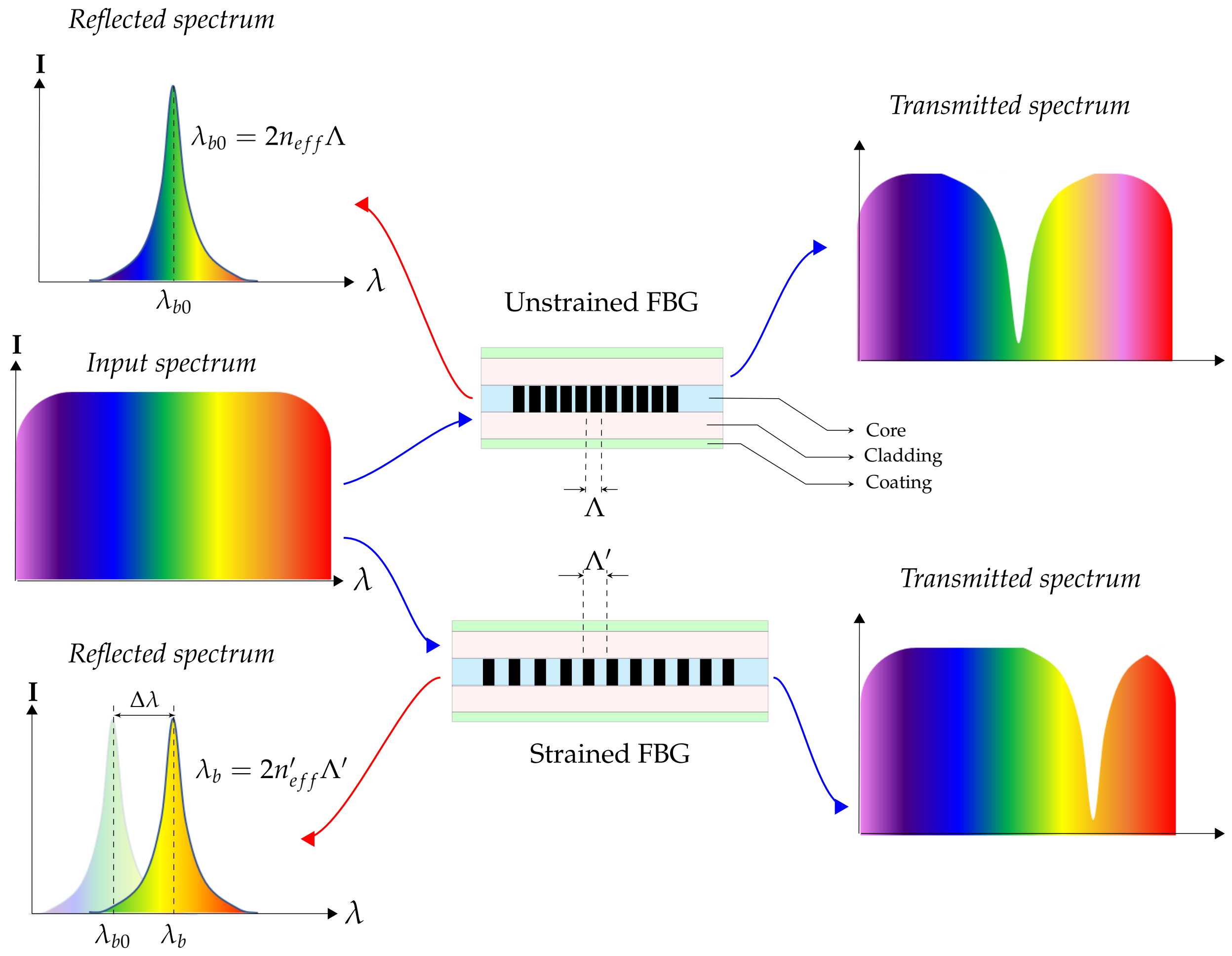

Figure 1: Working principle of FBG in an optical fibre.

Supplementary information 2. CBPM stiffness details

An overview of the Beam Constraint Model (BCM) for a simple beam flexure of uniform thickness and initially straight (non bent) is provided below. The BCM is based on the *Euler-Bernoulli (E-B) beam theory* with an assumption that the initially planar cross-sections remain planar and perpendicular to the neutral axis after deformation. These assumptions are applicable more generally to long and slender beams with variable cross-sections and general loading, for small bending deformations, around 10% of the beam length. A simple

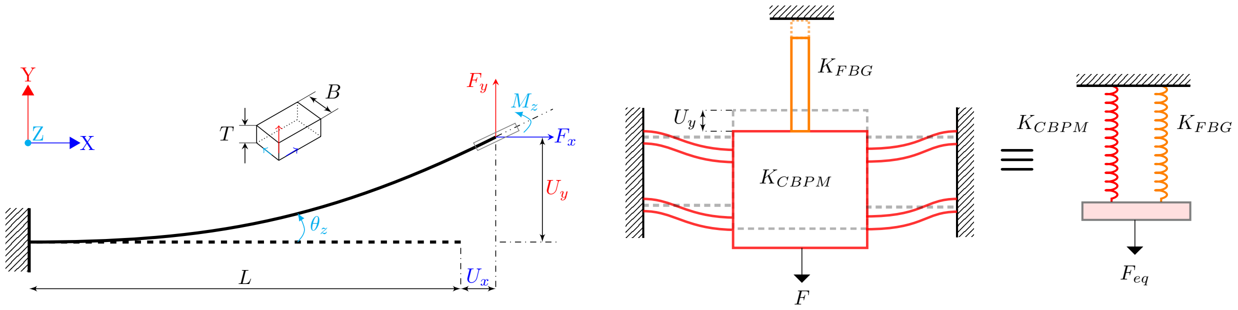

(a) **(b)**

Figure 2: (a) Deformation of a flexural beam; (b) Deformation of the four-arm CBPM.

beam with specific end load conditions is shown in Fig. 2 where L is the length, T is the thickness, B is the depth of the beam. F_x, F_y and M_z are the resulting end load displacements with respect to the co-ordinate frame X-Y-Z. Here, X is referred to as the axial direction, Y is referred to as the transverse direction, I_{zz} refers to as the second moment of area about the Z axis, E is the Young's modulus and ρ is the radius of curvature at a given cross-section.

Based on the assumption of the E-B beam theory, the following governing relation can be obtained:

$$\frac{E}{\rho(x)} = \frac{M_z(x)}{I_{zz}} \tag{3}$$

To generate the load-displacement relation from the governing relation, firstly, the bending moments have to be expressed in terms of the beam end loads (Eq. 4). Secondly, the beam curvature $\rho(x)$ has to be expressed in terms of the position co-ordinates and the displacement variable of beam (Eq. 5). Lastly, the load equilibrium and curvature expressions are substituted in governing equations to produce the beam's governing differential equation (Eq. 6).

$$M_z(x) = M_z + F_y(1 + U_x - x) - F_x(U_y - U_y(x)) \tag{4}$$

$$\frac{1}{\rho(x)} = y''(x) \tag{5}$$

By substituting Eq.4 and Eq. 5 for Eq. 3, it results that:

$$EI_{zz}U_y''(x) = M_z + F_y(1 + U_x - x) - F_x(U_y - U_y(x)) \tag{6}$$

Further, when differentiating twice w.r.t x:

$$U_y^{iv}(x) = \frac{F_x}{EI_{zz}} U_y''(x) \tag{7}$$

The beam equation is a fourth order differential equation and ought to satisfy the the displacement function (Eq. 7). It is also known that the beam equation requires only four boundary conditions as shown below:

1. Beam displacement at $x = 0 \rightarrow U_y(0) = 0$
2. Slope of beam displacement at $x = 0 \rightarrow U_y'(0) = 0$
3. Bending moment of the beam at $x = L \rightarrow U_y''(L) = \frac{M_z}{EI_{zz}}$
4. Shearing force of the beam at $x = L \rightarrow U_y'''(L) = \frac{-F_y + F_x U_y'(L)}{EI_{zz}}$

By applying the above four boundary conditions and solving the equation, there will be a linear relation between the end loads and displacements. But the closed form stiffness terms are not elastic (linear along the transverse loads and displacements) but transcendental functions of the axial load. These load displacement relations are too far complex to solve and approximate relations give a closed-form transverse direction load-displacement relation:

$$\begin{bmatrix} \frac{F_y L^2}{EI_{zz}} \\ \\ \frac{M_z L}{EI_{zz}} \end{bmatrix} = \begin{bmatrix} k_{11}^{(0)} & k_{12}^{(0)} \\ \\ k_{21}^{(0)} & k_{22}^{(0)} \end{bmatrix} \begin{bmatrix} \frac{U_y}{L} \\ \\ \theta_z \end{bmatrix} + \frac{F_x L^2}{EI_{zz}} \begin{bmatrix} k_{11}^{(1)} & k_{12}^{(1)} \\ \\ k_{21}^{(1)} & k_{22}^{(1)} \end{bmatrix} \begin{bmatrix} \frac{U_y}{L} \\ \\ \theta_z \end{bmatrix} \tag{8}$$

The approximation results in less than 1% error [2] since it is based on an infinite series expansion and truncation of the given transcendental function. For detailed Taylor series expansions of the actual transcendental functions and their algebraic approximations, reader is encouraged to read an article [2].

Next, the geometric constraint imposed by the beam arc length may be captured via the following integral, to determine the dependence of the axial displacement U_x on the transverse displacements:

$$L + \frac{1}{k_{33}} \frac{F_x L^3}{EI_{zz}} = \int_0^{L+U_x} \left\{ 1 + \frac{1}{2}(U_y'(x))^2 \right\} dx \tag{9}$$

The left and right-hand sides of this equation represent the beam length before and after the bending deflection, respectively. By using $U_y(x)$ solution for Eq. 7, Eq. 9 can also be

Table 1: Characteristic coefficients for beam [3]

$k_{11}^{(0)}$	12	$k_{11}^{(1)}$	$\frac{6}{5}$	$g_{11}^{(0)}$	$-\frac{3}{5}$	$g_{11}^{(1)}$	$\frac{1}{700}$	
								$k_{33} = \frac{12}{(T/L)^2}$
$k_{12}^{(0)}$	-6	$k_{12}^{(1)}$	$-\frac{1}{10}$	$g_{12}^{(0)}$	$-\frac{1}{20}$	$g_{12}^{(1)}$	$-\frac{1}{1400}$	
$k_{22}^{(0)}$	4	$k_{22}^{(1)}$	$\frac{2}{15}$	$g_{22}^{(0)}$	$-\frac{1}{15}$	$g_{22}^{(1)}$	$\frac{11}{6300}$	

solved to reveal the component of U_x that has a quadratic dependence on U_y and θ_z. A series expansion and truncation to the first power in F_x yields:

$$\frac{U_x}{L} = \frac{1}{k_{33}}\frac{F_xL^2}{EI_{zz}} + \begin{bmatrix} \frac{U_y}{L} & \theta_z \end{bmatrix} \begin{bmatrix} g_{11}^{(0)} & g_{12}^{(0)} \\ g_{21}^{(0)} & g_{22}^{(0)} \end{bmatrix} \begin{bmatrix} \frac{U_y}{L} \\ \theta_z \end{bmatrix} + \frac{F_xL^2}{EI_{zz}} \begin{bmatrix} \frac{U_y}{L} & \theta_z \end{bmatrix} \begin{bmatrix} g_{11}^{(1)} & g_{12}^{(1)} \\ g_{21}^{(1)} & g_{22}^{(1)} \end{bmatrix} \begin{bmatrix} \frac{U_y}{L} \\ \theta_z \end{bmatrix} \tag{10}$$

Eq. 8 and Eq. 10 are the constitutive equations of the BCM. The stiffness coefficient (k) and consraint coefficient (g) are non-dimensional beam characteristic coefficients that are solely dependent on the beams geometry and not on its absolute size. For a simple beam, these values are listed in Table 1 and they are referred to from a work [3]. Further, all loads, displacements and stiffness terms can be normalized with respect to beam parameters given by:

$$\frac{F_xL^2}{EI_{zz}} = f_x; \quad \frac{F_yL^2}{EI_{zz}} = f_y; \quad \frac{M_zL}{EI_{zz}} = m_z$$

$$\frac{U_x}{L} = u_x; \quad \frac{U_y}{L} = u_y; \quad \frac{T}{L} = t; \quad k_{33} = \frac{12}{t^2}$$

In a CBPM, each flexible beam is considered to be a fixed guided mechanism. Hence, we can substitute $u_x = 0$ and $\theta_z = 0$. Also, by substituting the values of the characteristic coefficient of the beam (tabulated in Table 1), we can evaluate:

$$f_x = \frac{6\ u_y^2}{\frac{10}{k_{33}} + \frac{u_y^2}{70}} \tag{11}$$

$$f_y = u_y(12 + 1.2f_x) \tag{12}$$

When a CBPM is displaced along the transverse direction, the input force required to produce the corresponding displacement will be four times the force for single beam in the transverse direction. This is due the fact that, when CBPM moves along transverse direction, displacement of four parallel beams occur simultaneously. Therefore, Eq. 12 becomes:

$$f = u_y(48 + 4.8f_x) \tag{13}$$

The stiffness in the transverse direction can be calculated by substituting in Eq.11 in 13 and by rewriting the equation as:

$$\frac{f_y}{u_y} = \frac{F_y L^3}{U_y EI} = 48 + \frac{5.76u_y^2}{\frac{10}{k_{33}} + \frac{u_y^2}{70}} \tag{14}$$

$$\boldsymbol{K_{CBPM}} = \frac{F_y}{U_y} = \left(48 + \frac{5.76u_y^2}{\frac{10}{k_{33}} + \frac{u_y^2}{70}}\right)\frac{EI}{L^3} \tag{15}$$

where K_{CBPM} is the transverse stiffness of the CBPM.

References

[1] Rao, Y.J.. In-fibre Bragg grating sensors. Measurement science and technology 1997;8(4):355.

[2] Awtar, S., Slocum, A.H., Sevincer, E.. Characteristics of beam-based flexure modules. Journal of Mechanical Design 2007;129(6):625–639.

[3] Awtar, S., Sen, S.. A generalized constraint model for two-dimensional beam flexures: nonlinear load-displacement formulation. Journal of Mechanical Design 2010;132(8).
